# Evaluation of potential factors compromising the use of electronic whiteboards 17 month after their implementation in Slagelse Emergency Department

**DOI:** 10.1186/1757-7241-21-S2-A5

**Published:** 2013-09-09

**Authors:** Stine Vestergaard Elbæk, Tim Løye Møller, Gustav From

**Affiliations:** 1 Emergency Department, Slagelse Hospital, Slagelse, Denmark; 2 Humanistisk Teknologisk Studie, Roskilde University, Roskilde, Denmark

## Background

Emergency departments (ED) have recently been established throughout Denmark, and their organization is still under transformation. The departments are characterized by a high turnover of staff. To ensure quality of care and efficiency of work electronic whiteboards (EW) have been implemented. The EWs create an overview allowing staff to assess who need care the most and to coordinate resources. May 2011 EWs were implemented at Slagelse ED, and a guideline for its use was written.

The aim of this study was to detect potential factors compromising the use of EWs in its operational phase 17 months after implementation.

## Methods

The study was designed as a qualitative study using observations and interviews to collect data.

November 2012 four external surveyors, students from University of Roskilde, made 20 hours of observations of physicians, nurses and secretaries during 3 days. 3 physicians (consultant, staff specialist, and junior doctor) and 3 nurses (leader, coordinating and clinical) were interviewed.

## Results

An inconsistency in the use of the EWs was observed causing challenges in the daily workflow. Disagreements on what should be registered, and by who and when were also observed.

Both physicians and nurses thought that the inconsistency in use was caused by two factors: firstly, the lack of a detailed mutually accepted guideline dictating who was responsible for the different functions and for registration of different types of information on the board, and secondly, limited introduction of the systems functionality to new staff.

## Conclusion

The study showed inconsistency in the staffs’ use of the EWs in its operational phase. The study suggested that this might be remedied by a regularly adjusted and mutually accepted guideline for use of EWs and a continuous thorough educational effort on new staff.

A clear guideline facilitates a better introduction and a better introduction contributes to implementation and maintenance of the guidelines, which means the two interventions enhance each other on the staffs’ use of the EW.

The study warrant further studies in Slagelse ED and EDs elsewhere to show if inconsistent use and outdated guidelines are widespread and to show if updated guidelines and education can stabilize the use of EWs.

